# A new method for mining information of co-expression network based on multi-cancers integrated data

**DOI:** 10.1186/s12920-019-0608-2

**Published:** 2019-12-30

**Authors:** Mi-Xiao Hou, Ying-Lian Gao, Jin-Xing Liu, Junliang Shang, Rong Zhu, Sha-Sha Yuan

**Affiliations:** 1 School of Information Science and Engineering, Qufu Normal University, Rizhao, China; 2 Qufu Normal University Library, Qufu Normal University, Rizhao, China; 3 Co-Innovation Center for Information Supply & Assurance Technology, Anhui University, Hefei, China

**Keywords:** Gene co-expression network, TCGA, Abnormally expressed genes, Mutual information, Pearson correlation coefficient

## Abstract

**Background:**

Gene co-expression network is a favorable method to reveal the nature of disease. With the development of cancer, the way to build gene co-expression networks based on cancer data has been become a hot spot. However, there are still a limited number of current node measurement methods and node mining strategies for multi-cancers network construction.

**Methods:**

In this paper, we introduce a new method for mining information of co-expression network based on multi-cancers integrated data, named PMN. We construct the network by combining the different types of relevant measures (linear and nonlinear rules) for different nodes based on integrated gene expression data of multi-cancers from The Cancer Genome Atlas (TCGA). For mining genes, we combine different properties (local and global characteristics) of the nodes.

**Results:**

We uncover more suspicious abnormally expressed genes and shared pathways of different cancers. And we have also found some proven genes and pathways; of course, there are some suspicious factors and molecules that need clinical validation.

**Conclusions:**

The results demonstrate that our method is very effective in excavating gene co-expression genes of multi-cancers.

## Background

In recent years, the study of molecular biology networks has provided a powerful tool for exploring complex life activities. With the diversity and high dimension of biological sequencing data, the advantages of network pattern mining become more and more noticeable. The network can macroscopically construct the connection between biomolecules, which can enrich the amount of excavation information. Gene expression data reflect the abundance of the directly or indirectly measured gene transcription product by mRNA in cells. These data can be utilized to analyze genes that have changed their expression and the relationship between them. And gene expression data also imply how the activities of genes are affected under different conditions. Constructing co-expression networks based on gene expression data is a universal approach to molecular biology networks. There are two main types of patterns based on gene expression data: regard genes as nodes or samples as nodes. Typical representatives such as Weighted Gene Co-expression Network Analysis (WGCNA) [1] and Similarity Network Fusion (SNF) [2]. In this paper, our improvements are based on nodes for network construction.

The similarity measures based on gene expression profiles during the process of network construction are mainly obtained by calculating the linear or non-linear correlation coefficient of different gene expression profiles. Among them, universal linear correlation measurements include Pearson Correlation Coefficient (PCC), Spearman Correlation Coefficient (SCC), and Partial Correlation Coefficient which exclude other variables and so on. Measurements of non-linear correlations include, for example, Mutual Information (MI) and so on. Among them, PCC and MI are more commonly utilized. WGCNA adds an index to the PCC so that the distribution of correlation coefficient values gradually accords with the scale-free distribution. WGCNA is a method of mining module information from sequencing data. WGCNA is the current effective method with higher recognition. But it is based on the module method, lack of directional mining for the node. Butte and Kohane firstly used MI as a measure of correlation between the genes and constructed an MI genes-related network [3]. Since then, the application of MI has been widely up [4]. There are no specific standards for the association measure, and some scholars have compared above two types of measures and found that each has its own advantages and disadvantages [5].

In terms of data, the current gene network is primarily focused on single-disease dataset. With the development of next generation sequencing technology, the production of a large number of multi-omics data provides a new direction for disease research scholars to study [6, 7]. Multi-omics data can be better carried out examination research by integrating variety datasets. Integrated data include the integration of different data types of the same disease, as well as data integration of diverse diseases. These data have important biological significance for discovering the pathogenesis of diseases and providing rich and implicit information. The data from The Cancer Genome Atlas (TCGA, https://cancergenome.nih.gov/) are more interested in the changes in gene expression of human cells after birth (acquired variation). Most of the datasets provided by TCGA are integrated data, multi-group data. In addition, there are few ways to build a network based on integrated data of multi-cancers to further exploit causative agents, aiming to find the common disease-causing pathways or biotic factors in multi-cancers. In the most process of network constructions, the definition of measure between nodes is single, linear or non-linear. In the node mining aspect, the node evaluation has yet to be improved.

Based on the integrated gene expression data of the three types of cancers from TCGA, our paper provides a novel idea of constructing a co-expression network and attempts to discover common disease-causing modules of three kinds of cancers so as to provide some references for the research and development of cancers. The procedure of constructing a network draws on the combination of linear (PCC) and nonlinear (MI) similarity between genes at the same time which is called PMN, eliminating the single association of network nodes and enriching the information contained in gene networks. Considering the local and global effects in node evaluation, it is helpful for the comprehensive exploitation of nodes.

## Methods

In this section, we will introduce the steps of PMN, including network construction and strategies of evaluating nodes.

The PCC between two genes *X* and *Y* is defined as follows:

1 $$ {\mathrm{PCC}}_{XY}=\frac{\sum_i^n\left({X}_i-\overline{X}\right)\left({Y}_i-\overline{Y}\right)}{\sqrt{\sum_{i=1}^n{\left({X}_i-\overline{X}\right)}^2{\sum}_i^n{\left({Y}_i-\overline{Y}\right)}^2}}, $$

where *X*_*i*_ and *Y*_*i*_ represent the observations of the gene *X* and *Y* on the *i* ‐ th sample, respectively. *n* is the number of samples, $\overline{X}$ and $\overline{Y}$ are the mean observations on the gene *X* and *Y* in all samples.

The entropy of discrete random variables is defined as follows:

2 $$ H(X)=-\sum \limits_xp(x)\log p(x), $$

where *p*(*x*) = Pr {*X* = *x*} is the probability density function, the mutual information between two genes *X* and *Y* can be described as:

3 $$ MI\left(X,Y\right)=H(X)+H(Y)-H\left(X,Y\right), $$

where *H*(*X*, *Y*) =  − ∑_*x*_∑_*y*_*p*(*x*, *y*) log *p*(*x*, *y*) is joint entropy. Many studies have shown that MI is better for discrete values [8]. But gene expression data contain all continuous values. In order to make better use of the MI measure, we learn from the treatment in Minet [8] method to discretize continuous values.

The relationship between genes is not unique and the existing single measure is difficult to accurately describe the relationship between genes. We consider the different relationships between genes, so that the stronger relationships can be retained in the networks. The definition of adjacency matrix *A* = [*a*_*ij*_] is based on the construction of two measures to retain the linear or non-linear relationships of genes:

4 $$ {a}_{ij}=\left\{\begin{array}{l}1\kern1.8em if\kern0.4em PCC>{t}_1\kern0.5em or\kern0.5em MI>{t}_2,\\ {}0\kern1.6em else,\end{array}\right. $$

where *t*_1_ and *t*_2_ are the filter thresholds of the matrices about PCC and MI. Theoretically, there is no strict requirements to formulate *t*_1_ and *t*_2_ .

For node mining, on the one hand, degree centrality of node *D*_*x*_ is taken into consideration: degree centrality is the most basic topological property that describes a single node in the network. The degree centrality of node *v* refers to the number of edges connected directly to *v* in the network, implying the local characteristics of a node. On the other hand, the betweenness centrality of a node is also excavable for mining nodes. The betweenness of a node is a measure of the sum of its proportions appearing in the shortest path between other nodes. The betweenness of the node is defined as follows:

5 $$ {B}_x={\sum}_{i\ne x\ne j\in X}\frac{\sigma_{ixj}}{\sigma_{ij}}, $$

where *σ*_*ij*_ represents the counts of shortest path between node *i* and *j*, but *σ*_*ixj*_ is the number of shortest path via node *x*. The betweenness indicates the role a node plays in connecting other nodes to each other. The higher the betweenness, the more important the node is in maintaining tight connectivity for networks, which reflecting the global characteristics of a node.

In this paper, we define a weighted value *W*_*x*_ for each gene node in the network by combining above two aspects to select the abnormally expressed genes:

6 $$ {W}_x={D}_x\times {B}_x. $$

**Fig. 1:**
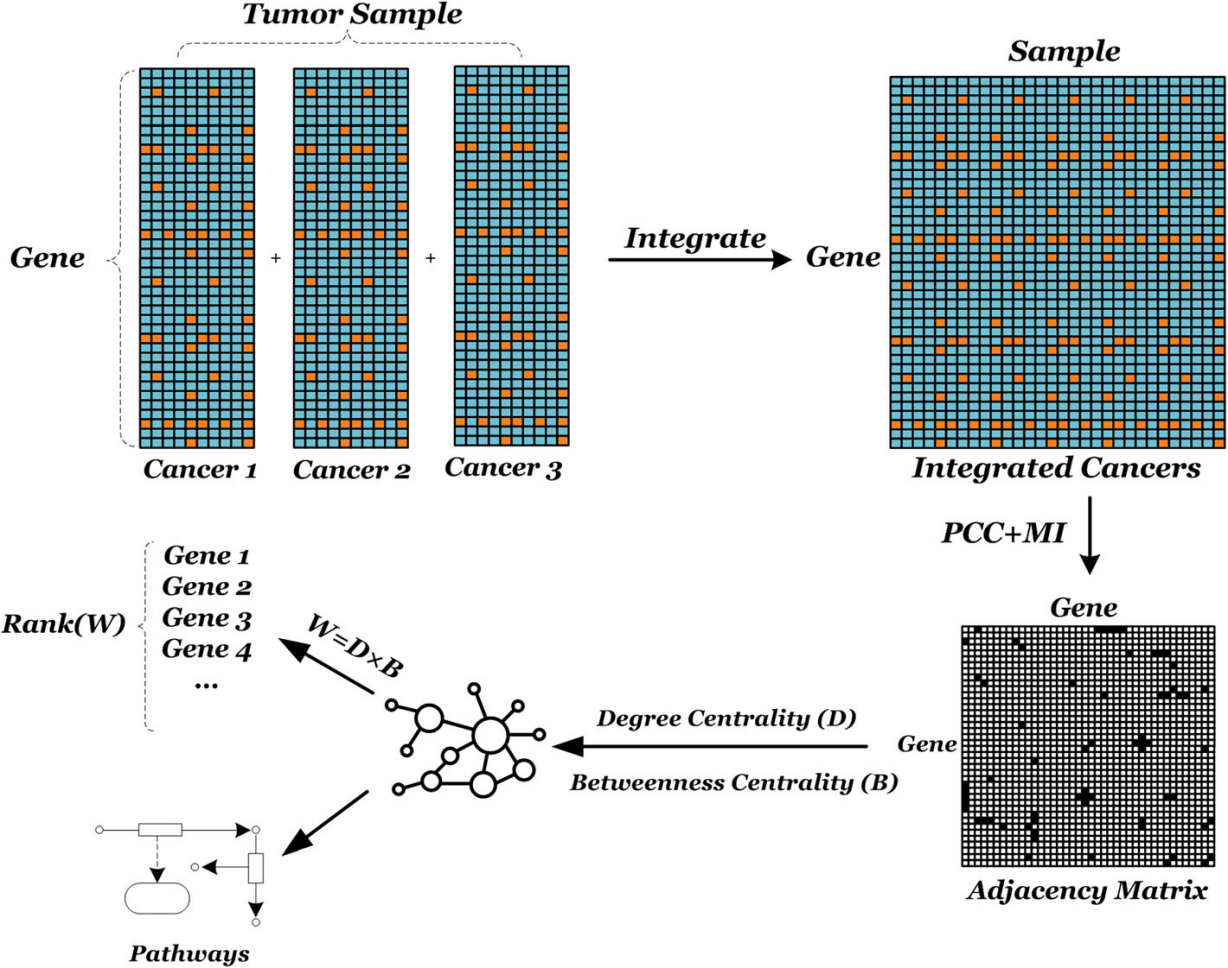
Diagram of PMN for mining information on multi-cancers integrated data

Taking the type of values into account, multiplication can better reflect the effect of two characteristics. The measured values of the degree are greater than 0, and the values of the betweenness are less than 1. Considering the type of values and conducting extensive experimental tests, multiplication can better reflect the effect of two characteristics. It is also a combination of local and global features for nodes. Detailed schematic diagram of the overall process flow shown in Fig. 1, the following is a detailed process and details.

## Experiments

### Datasets

The experimental data are cancer integrated gene expression data of three types of cancer from the TCGA (RNA-seq data at level 3) database: Pancreatic adenocarcinoma (PAAD), Esophageal carcinoma (ESCA) and Head and Neck squamous cell carcinoma (HNSC). There are more than 20,000 genes in each dataset, and the samples are different from each other. Among them, HNSC contains 398 tumor samples, ESCA 183 contains tumor samples, and 176 tumor samples are from PAAD. After removing the missing values, we integrated them into a matrix which contains 757 samples with 15,442 features for gene correlation calculation.

### Threshold selection

**Fig. 2:**
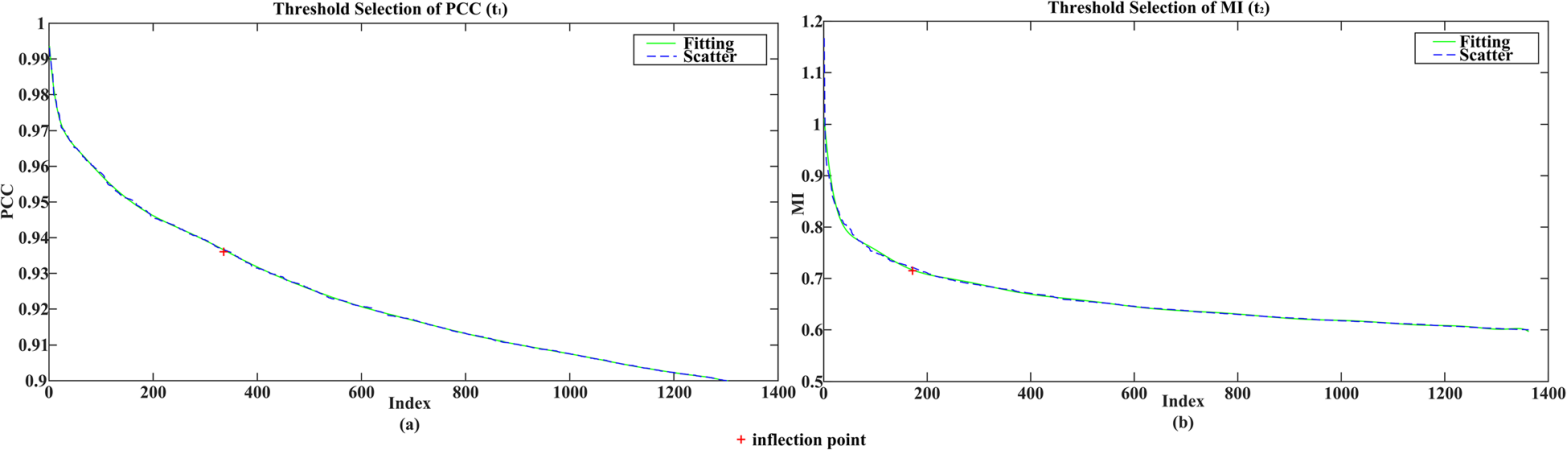
Inflection point selection

Values of PCC correlation matrix are in the range of [0, 1], and correlation values of the MI matrix have no specific scope. Taking into account the overall situation, after all the correlation values of the two matrices are sorted, curve fitting is performed, and then the first inflection point is selected separately as thresholds (*t*_1_ = 0.9363 and *t*_2_ = 0.7271) of the MI and PCC, as is shown in Fig. 2 (red cross symbol is inflection point). Since there are plenty of correlation values and in order to facilitate the display, we only intercepted the previous points to exhibit.

## Results

**Fig. 3:**
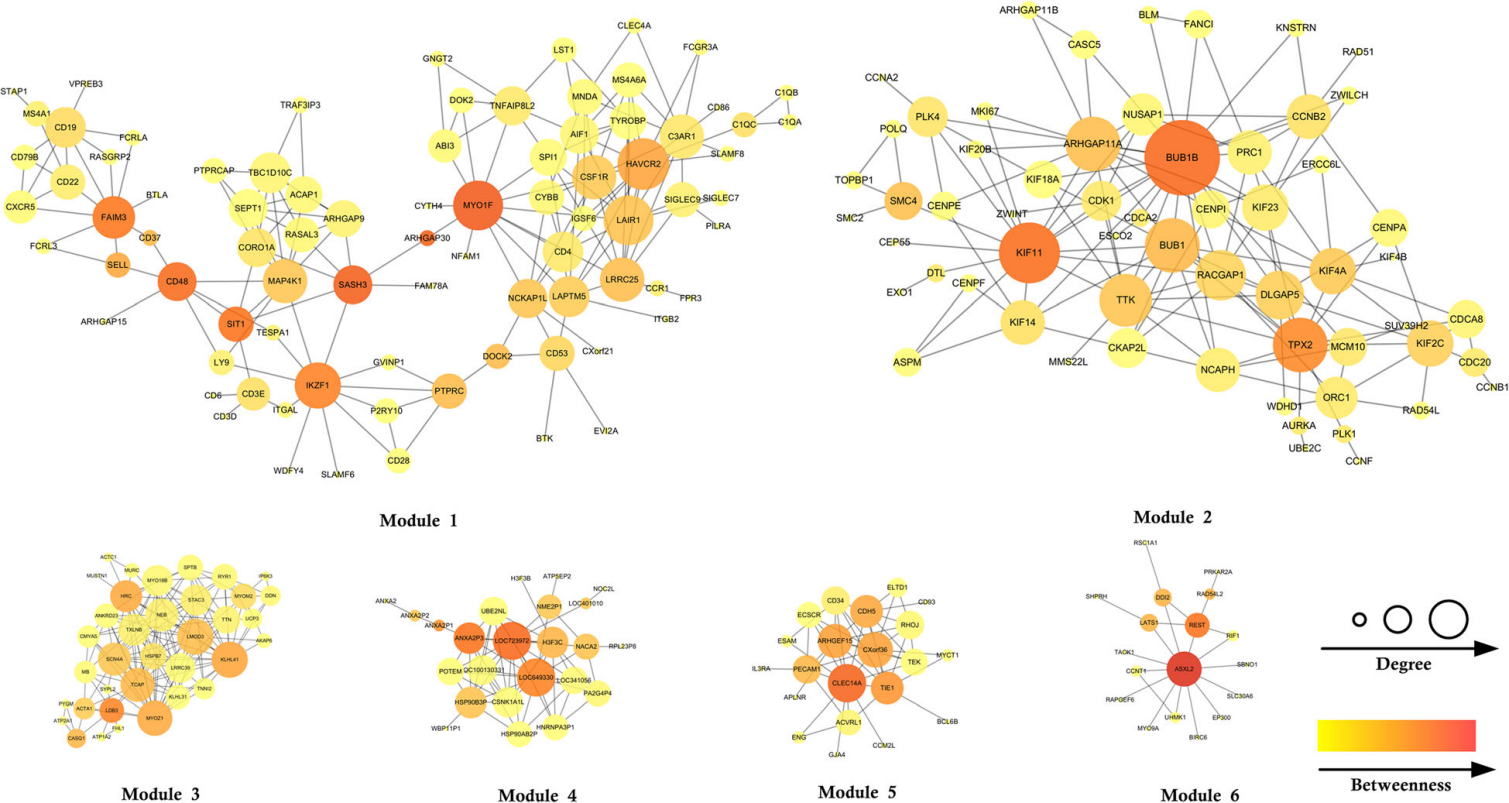
Networks of 6 Modules under PCC and MI

After threshold filtering, we can get 242 gene nodes and 6 modules and each interaction group covers at least 18 nodes, which are visualized by Cytoscape [9], as is illustrated in Fig. 3. Due to too few genes in other modules, we cannot calculate the relative role of nodes, and we will not consider them here. In the Fig. 3, the size of a node indicates the degree of a node, and the color depth indicates the size of a messenger. Through the network of nodes in the interrelationship, we can obtain the enrichment of pathways for each module. The connections between the nodes indicate the correlation between genes, strictly dependent on PCC and MI. The discovered node interaction group in the Fig. 3 contains fewer nodes, mainly due to the threshold selection method. Threshold inflection point selection method is also a truncated threshold selection strategy; this strategy will lead to the network more serious block. And in the networks, related and unrelated nodes become straightforward, interactive modules bound not too much. There are also levels of correlations (differences in connectivity for each node) in our networks. Although in many genes, only 1% of the information about gene interactions is retained, it is from another perspective that these retainable modules must be highly relevant modules. It should be more effective to analyze the role of nodes in these highly correlated and well-structured modules.

Finally, genes with larger weighted values implied a higher importance in the interaction group, and we list the top 10 for analysis, genes are listed in Table 1. The corresponding icon can also be found in Fig. 3. The corresponding notes are available on the GeneCards (http://www.genecards.org/).

**Table 1:** The top 10 genes with higher weights

Gene	Summary
**ASXL2**	This gene encodes a member of a family of epigenetic regulators that bind various histone-modifying enzymes and are involved in the assembly of transcription factors at specific genomic loci.
**BUB1B**	This gene encodes a kinase involved in spindle checkpoint function.
MYO1F	The protein encoded by this gene is an unconventional myosin that may be involved in the intracellular movement of membrane-enclosed compartments.
**CLEC14A**	This gene encodes a member of the C-type lectin/C-type lectin-like domain (CTL/CTLD) superfamily. This family member plays a role in cell-cell adhesion and angiogenesis. It functions in filopodia formation, cell migration and tube formation.
**KIF11**	This gene encodes a motor protein that belongs to the kinesin-like protein family.
SASH3	The protein encoded by this gene contains a Src homology-3 (SH3) domain and a sterile alpha motif (SAM), both of which are found in proteins involved in cell signaling.
KLHL41	The encoded protein by this gene contains a BACK domain, a BTB/POZ domain, and 5 Kelch repeats. This protein is thought to function in skeletal muscle development and maintenance.
**MYOZ1**	The protein encoded by this gene is primarily expressed in the skeletal muscle, and belongs to the myozenin family.
**TPX2**	This gene is a Protein Coding gene. Diseases associated with TPX2 include Hepatocellular Carcinoma
**CD48**	This gene encodes a member of the CD2 subfamily of immunoglobulin-like receptors which includes SLAM (signaling lymphocyte activation molecules) proteins.

The bold data are cancer-related genes

According to the proven material, ASXL2 is related to cancer in several tissue types, such as breast, bladder, pancreas, ovary, prostate, and blood. This gene plays a significant role in neurodevelopment, cardiac function, adipogenesis, and osteoclastogenesis; the latest studies have shown that reproductive mutation gene ASXL2 in the maintenance of normal hematopoietic stem cell function of Acute Myeloid Leukemia (AML) has antitumor effect [10]. Abnormal expression of BUB1B has turned out to be associated with the development of lung adenocarcinoma, breast cancer, colorectal cancer and prostate cancer [11–13]. CLEC14A is considered as a candidate for tumor vascular targeting due to its presence at higher levels in tumor endothelium than in normal tissue endothelium. KIF11 abnormalities in prostate cancer, ovarian cancer were found [14, 15]. Some studies have shown that MYOZ1 has correlation with oral squamous cell carcinoma and it could be regarded as a molecular target for cancer applications. The over-expression of detection for TPX2 can be used as a prognostic indicator and therapeutic target for gastric cancer, prostate cancer, cervical cancer and so on [16–18]. Some scholars detected a decrease in CD48 expression in hepatocellular carcinoma cells [19].

The relevant pathways are found in 6 modules and we take the top 10 by p-values as is listed in the Table 2. Pathway enrichment databases involved: Kyoto Encyclopedia of Genes and Genomes (KEGG) and Reactome. *Cell adhesion molecules (CAMs)* play an important role in the tumorigenesis of cervical cancer [20]. The up-regulation of cyclin B1/Cdc2 plays a pivotal role in treated breast cancer cells in human in stagnation of *Mitotic Prometaphase* [21]. In the study of *Staphylococcus aureus infection*, patients with malignant tumors have a high rate of *S. aureus* infection [22]. The changes in the cancer cells of *MHC class II antigen presentation* have been noticed. Correspondingly, the development of efficient vaccines to activate the immune system against cancer has been investigated [23]. Over-expression of *FOXM1 transcription factor network* is linked to the development and pathogenesis of cervical cancer [24].

**Table 2:** Cancer related suspicious pathways

Gene Set	*P*-value	FDR
Mitotic Prometaphase(R)	1.11E-16	4.22E-14
Signaling by Rho GTPases(R)	2.31E-14	4.39E-12
Aurora B signaling(N)	1.81E-11	2.28E-09
PLK1 signaling events(N)	4.92E-11	4.67E-09
Mitotic Metaphase and Anaphase(R)	2.17E-10	1.65E-08
FOXM1 transcription factor network(N)	1.23E-07	7.77E-06
Cell adhesion molecules (CAMs)(K)	1.66E-07	8.96E-06
Hematopoietic cell lineage(K)	1.36E-06	6.34E-05
Staphylococcus aureus infection(K)	1.51E-06	6.34E-05
MHC class II antigen presentation(R)	4.17E-05	9.18E-04

In order to further prove the advantages of PMN, we compare it with WGCNA. Firstly, we compare the number of pathways that found in the networks built by PMN and WGCNA under same scale (the number of nodes is almost, WGCNA: 230, PMN: 242), as shown in Table 3. It is clear that the network constructed by PMN which can find more pathways. And in order to understand more about these pathways, we contrast the *p*-values of the common pathways of the more relevant network modules obtained by the two methods. In the common pathways between the two, the difference about *p*-values is still quite significant, as is listed in Table 4.

**Table 3:** Comparison for pathways of PMN and WGCNA

Method	Number of pathways
PMN	380
WGCNA	260

**Table 4:** Comparison for *p*-values of common pathways found by PMN and WGCNA

GeneSet	PMN		WGCNA	
	*P*-value	FDR	*P*-value	FDR
Signaling by Rho GTPases(R)	2.31E-14	4.39E-12	0.7673	0.7673
Cell adhesion molecules (CAMs)(K)	1.66E-07	8.96E-06	0.7055	0.7055
Factors involved in megakaryocyte development and platelet production(R)	4.11E-06	1.40E-04	0.6038	0.6038
TCR signaling in naïve CD4+ T cells(N)	5.57E-06	1.73E-04	0.4304	0.4304
Neutrophil degranulation(R)	1.12E-05	3.24E-04	0.4188	0.4203
Cell Cycle Checkpoints(R)	2.28E-05	5.24E-04	0.7494	0.7494
Intra-Golgi and retrograde Golgi-to-ER traffic(R)	1.85E-04	3.02E-03	0.6953	0.6953
Oocyte meiosis(K)	1.94E-03	0.0213	0.0207	0.4203
Progesterone-mediated oocyte maturation(K)	2.72E-03	0.0278	0.0496	0.4203
Cell surface interactions at the vascular wall(R)	2.79E-03	0.0279	0.5218	0.5218

The genes found by our method can match the pathways more accurately and with a lower error rate. WGCNA differs from PMN in that WGCNA is a method that focuses on module analysis, whose all modules together cover all genes, and focusing on the overall contribution of each module. In each module of WGCNA, the interactions of genes are very close and the ways of node mining is single. It is not conducive to distinguish the role of nodes in the single module. In principle, our method only retains some of the more relevant modules, focusing on the role of genes within the modules.

## Discussion

The abnormally expressed genes have different statuses in cell networks and may have different effects on the network. Different network structures also have different tolerances for gene mutations. In this sense, the tumor is actually a kind of molecular network disease. So, it will be one-sided to ignore the whole effects and emphasize the role of single gene. PMN utilizes network structure to simulate gene interactions of cancers and discovers some abnormally expressed genes in the node relationships of networks, which have a significant effect in digging out co-expressed abnormally genes of multi-cancers.

As a kind of biomolecule network, the co-expression network has the nature of scale-free: most nodes have a small number of connections, and a small number of nodes have a large number of connections. This characteristic shows that in the dynamic process possessed by the biomolecular network, a few molecules represented by the nodes play a key role. PMN also follows this rule and takes the mining of key pathogenic nodes as the primary goal. Based on the global and local characteristics of the nodes, more suspicious abnormal genes are detected.

Among the top genes found by the PMN, ASXL2, BUB1B, KIF11 and TPX2 have been clinically proven to be associated with multi-cancers, which are a variety of genes commonly identified as mutated in cancers. The genes that abnormally and simultaneously expressed in multi-cancers are naturally considered to be key genes for the development of malignant tumors. These genes are of great significance for the prognosis of other cancers. MYO1F, SASH3 and KLHL41 are the candidate pathogenic genes that shared with multi-cancers in this paper. Until now, no research has shown that the improper expression of these genes may cause consequences for canceration, but it can still provide clues for clinical medicine. The progress of the three cancer research is not particularly impressive; we found some common anomalies of cancers that went well with the research by building networks based on the integrated dataset. To some extent, it will be beneficial for the study of three kinds of cancer.

Although the linear association rules can describe the relationship between most of the nodes, it cannot deny that the non-linear relationship exists in the gene expression data in various forms. Therefore, MI is a supplement to PCC, which makes the structure of network more complete. Degree centrality of a node is a very important symbol in the network, but simply considering the degree is one-sided. Betweenness centrality measures the effect of a node on the tightness of the entire network from a global perspective. The combination of the two can detect more critical nodes to some extent. For example, both KIF11 and CD48 are nodes that are less prominent in connectivity and betweenness, but the weighted values are prominent, indicating that the weighted is reasonable.

On the network scale, our approach only preserves a handful of the key modules of gene interaction, also gives the strategy of node mining in the module. It eliminates the influence of irrelevant information in high-dimensional data. And to a certain extent, it has some advantages for extracting key information.

## Conclusions

In this paper, we introduce a method of constructing a network that combines the linear (PCC) and non-linear (MI) measurements between genes, which is based on gene expression integrated data of three kinds of cancers from TCGA. Network construction eliminates the single association between network gene nodes and enriches the information contained in the gene network. At the same time, considering the degree and betweenness of the node from the local and global perspective on the evaluation of nodes, it is conducive to mining more critical abnormally expressed node in the entire network. By obtaining genes and pathways shared by several common cancers, the effectiveness of this approach is demonstrated. Besides, the availability of additional genes and pathways may provide evidence for cancer research. Of course, the method of this article also has a lot to be improved. For example, gene expression data itself carry some noise, which will bring some interference to the latter network. Whether the weighting values of nodes can be obtained by a better combination of their characteristics, which is also the focus of our next study.

## Data Availability

The datasets that support the findings of this study are available in https://cancergenome.nih.gov/.

## Abbreviations

ESCA: Esophageal carcinoma
HNSC: Head and Neck squamous cell carcinoma
MI: Mutual Information
PAAD: Pancreatic adenocarcinoma
PCC: Pearson Correlation Coefficient
TCGA: The Cancer Genome Atlas
WGCNA: Weighted Gene Co-expression Network Analysis
